# PaxDb 5.0: Curated Protein Quantification Data Suggests Adaptive Proteome Changes in Yeasts

**DOI:** 10.1016/j.mcpro.2023.100640

**Published:** 2023-08-31

**Authors:** Qingyao Huang, Damian Szklarczyk, Mingcong Wang, Milan Simonovic, Christian von Mering

**Affiliations:** Department of Molecular Life Sciences and Swiss Institute of Bioinformatics, University of Zurich, Zurich, Switzerland

**Keywords:** protein abundance, proteomics, mass spectrometry, proteome evolution, database

## Abstract

The “Protein Abundances Across Organisms” database (PaxDb) is an integrative metaresource dedicated to protein abundance levels, in tissue-specific or whole-organism proteomes. PaxDb focuses on computing best-estimate abundances for proteins in normal/healthy contexts and expresses abundance values for each protein in “parts per million” in relation to all other protein molecules in the cell. The uniform data reprocessing, quality scoring, and integrated orthology relations have made PaxDb one of the preferred tools for comparisons between individual datasets, tissues, or organisms. In describing the latest version 5.0 of PaxDb, we particularly emphasize the data integration from various types of raw data and how we expanded the number of organisms and tissue groups as well as the proteome coverage. The current collection of PaxDb includes 831 original datasets from 170 species, including 22 Archaea, 81 Bacteria, and 67 Eukaryota. Apart from detailing the data update, we also present a comparative analysis of the human proteome subset of PaxDb against the two most widely used human proteome data resources: Human Protein Atlas and Genotype-Tissue Expression. Lastly, through our protein abundance data, we reveal an evolutionary trend in the usage of sulfur-containing amino acids in the proteomes of Fungi.

## Highlights

• PaxDb offers reference protein abundance data for an unrivaled collection of species.
• The PaxDb human dataset aligns with benchmarks derived from two leading resources.
• PaxDb data unveils a consistent evolutionary trend of sulfur avoidance in yeast.

## Graphical Abstract

**Figure:**
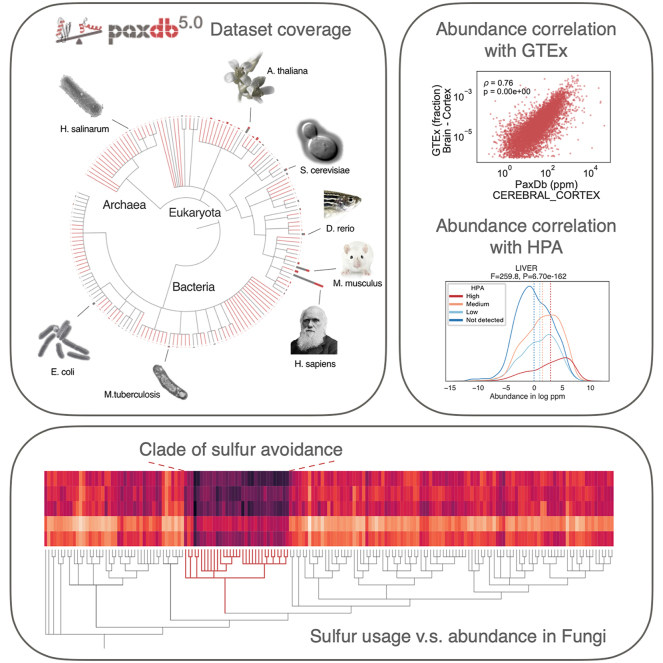

## In Brief

PaxDb is a comprehensive metaresource of protein abundance data across multiple organisms and tissues. Here, we describe the latest version 5.0, for which we have improved data standardization and expanded the number of individual datasets, organisms, and tissue groups, as well as the overall proteome coverage. We also present a comparative analysis of PaxDb human tissue proteomes against HPA and GTEx. Lastly, using PaxDb as reference, we uncovered a previously undescribed evolutionary trend in the amino acid composition in Fungi.

Biological processes are regulated at multiple levels. Although many cellular changes are clearly detectable already at the transcriptome level, it is the protein level that most accurately reflects the cellular state since proteins act as the direct executors of biological functions. Apart from a protein’s expression level, further regulatory potentials lie in its posttranslational modifications, subcellular localizations, and biological contexts. In a complex multicellular organism with a system of coordinated organs, protein expression patterns largely conform to the specific requirements and activity of the tissue or organ. Furthermore, protein expression profiles can differentiate between healthy and disease states, providing important markers and targets for diagnosis and treatment.

Thus, systematic measurements of protein expression levels facilitate both the understanding of fundamental biological processes and the design of new therapeutic strategies. Proteomics data collections have seen exponential growth in the last decade. Along with the data growth, analytical instrumentation and data processing methodologies for quantitative proteomics have rapidly progressed. Mass-spectrometry–based measurements provide the bulk of protein quantifications, with multiple workflows and modalities from stable isotope labeling to label-free quantification, from targeted, data-dependent acquisition (or shotgun) to data-independent acquisition modes, and involving multiple ion trap technologies—time-of-flight (1), linear quadrupole ion trap (2), and Orbitrap (3)—in terms of instrument configuration. For downstream data processing, a number of quantification software packages evolved with multiple pipelines to tackle different challenges in each experiment set-up, with the most prominant ones being MaxQuant (MQ) (4) and Proteome Discoverer (Thermo Fisher Scientific). A plethora of file formats are produced; input and output data at several levels of processed information are recorded in various forms with overlapping information content (5). Despite efforts to create a unified data standard with mzML (6) and mzTab (7), the legacy of viable file formats continues to create challenges for integrating and standardizing the existing data.

The PaxDb database (Protein Abundances Across Organisms) is an integrative metaresource dedicated to absolute protein abundance levels in whole organism or tissue-specific proteomes (8, 9). PaxDb focuses on creating a consensus view on normal/healthy proteomes and expresses abundance values in “parts per million” (ppm) in relation to all other protein molecules in the sample. Since the last PaxDb update, the proteomics community has grown continuously: roughly 1000 projects per month are submitted to ProteomeXchange, the largest centralized platform for MS-derived primary data submission (10), involving PeptideAtlas (11), PRIDE (12), iProX (13), and jPOST (14) among others. For the latest version 5.0 of PaxDb, we have further improved data integration by extending the types of raw data imported from the various repositories and by expanding the number of organisms and tissue groups as well as the proteome depth of previously covered organisms.

Using earlier versions of PaxDb as a reference, scientists have already modeled fundamental biological processes (15, 16, 17, 18, 19), formed hypothesis about stoichiometry in complexes (20, 21), studied tissue-specific functionalities (21, 22, 23), and verified new MS techniques and methodology (24, 25). Indeed, the overall protein abundance landscape is likely reflecting a fundamental, cross-species, structural and functional equilibrium (8). Proteins at the high end of the abundance distributions are particularly informative for evolutionary studies: their synthesis brings a significant cost to the organism, and they are observed to be coded more compactly, to have fewer introns, and to be subject to heavier codon optimizations (26, 27, 28, 29). As the biosynthetic energy costs of the various amino acids differ by as much as 7-fold, energetic effects—but also nutrient and element availability—shape the general direction of amino acid evolution (30, 31, 32). Episodes of nitrogen (N) limitation likely have lead plants to reduce the overall nitrogen presence in their proteome as compared with animal proteomes (33). Iron (Fe) limitation prompted most marine organisms to develop an iron-free version of ferredoxin, flavodoxin, as an electron transfer agent in their biochemical reactions (34, 35), and in extreme environments, a clade of *Procholorococcus* permanently lost ferredoxin in addition to losing 10% Fe containing proteins (36). Sulfur (S) is somewhat less studied. It is present in only two amino acids’ side chains, cysteine and methionine. Nevertheless, it has been shown that the effect of a single amino acid substitution involving sulfur is visible to selection in more than half of the proteome in a yeast model (37). While previous studies reached their conclusion through observations in a limited number of species and proteomics datasets, the PaxDb resource has the advantage of its large collection of protein abundance data with associated orthology relationships. Here, we use these data to present evidence of a strong and wide-spread sulfur avoidance at evolutionary timescales, in an entire clade of Fungi species.

## Experimental Procedures

### PaxDb Data

All data contained in PaxDb are derived from public repositories, open-access publications, or publicly accessible data supplements.

#### Abundance Data Inherited From PaxDb v4

Protein records in PaxDb are generally based on the same genome versions and identifier namespaces as those in the STRING database (38), including one-to-one mappings to Uniprot IDs. PaxDb v4 corresponds to STRING v10.0, whereas the updated PaxDb v5.0 corresponds to STRING v11.5 (38). Wherever genome annotations and protein sequences change between major PaxDb releases, the corresponding protein records are remapped to the newest annotation.

For datasets quantified by spectral counting, a recomputation is performed on the updated species’ complete proteome. For datasets consisting of protein identifiers and abundances, the protein names are remapped to the latest name-spaces using the identifier collections maintained by STRING, which provide identifier mappings for a total of 278 identifier systems.

#### Dataset Collection

Since 2014, the ProteomeXchange Consortium (10, 39) has become a centralized platform for MS proteomics data sharing. The selection of projects to be imported into PaxDb is based on project metadata and text mining of publications. The metadata of the projects were downloaded through the ProteomeXchange API, including project ID, species, year, keywords, list of files with extension, among other information. A full-text search was performed on all PubMedCentral publications, and those containing identifiers starting with PXD or RPXD were retrieved. Combining the project metadata and publication information, the relevant projects were manually selected with priority for highly cited publications and organisms not yet included in PaxDb. Keywords involving disease conditions, subcellular compartments, exclusively posttranslational modification or protein identification, virus, secretome, interactome, and metabolome were excluded.

During the project selection, the abundance data were downloaded from the supplementary tables in 98 publications.

An additional 421 project IDs were selected, and their files were downloaded from ProteomeXchange. The file extensions which were further processed included csv, xls/x, mzid, mgf, mzML, mzTab, msf, and txt.

#### Protein-Centric Data Processing

From publication supplements, abundance reports already aggregated to the protein level were mapped to the common identifier space using global alias file from STRING database. To recover any unmapped entries, additional steps were taken. In particular, protein IDs in the International Protein Index namespace (closed with its last update in September 2011) system were converted to UniProt ID with the mapping file (last release 2014-01) from UniProt Archive (UniParc). The converted Uniprot IDs as well as other unmapped IDs were searched through the NCBI E-utilities (40) to fetch their protein sequences. These sequences were blasted against the updated species proteomes and mapped *via* reciprocal best-hit matching (requiring a minimum 90% sequence identity in both directions). For datasets reporting protein abundances in the form of ortholog IDs from a closely related species (because mass spectrometry libraries and databases were available there), protein records were also blasted against the original species’ proteome and mapped *via* reciprocal best-hit matching.

In bulk-download files from ProteomeXchange, most protein-centric data are in the MQ output format “proteinGroup.txt” (4). Any “CON_” (contaminant) or “REV_” (reversed sequence) entries were excluded from protein Groups files, while all other protein names were mapped as described above. The intensity value for each protein entry was extracted, and the molecular weight and the theoretically observable peptides were calculated from the protein sequence. Then, the intensity-based absolute quantification was calculated, using Method #4 described in (41). Specifically, the relative abundance of a given protein was calculated by dividing the sum of its precursor peptide intensities by the count of peptides theoretically observable from a complete trypsin digestion of the protein sequence.

#### Peptide-Centric Data Processing

Wherever available, peptide-centric data were preferred over protein-centric data, and peptide-intensity data were preferred over peptide-count data, for all downstream data processing. From msf files, target and decoy peptides were separated. The false discovery rate score threshold was set to 0.01 to filter the valid target peptides. The peptide intensities were extracted for further processing. All imported peptide data were then further processed with the pipeline described in study by Weiss *et al.* (42). In detail, the relative abundance of a given protein was determined by normalizing the sum of each constituent peptide's quantity (peak intensity or spectral counts)-length product by the total corrected length of theoretically observable peptides.

#### Data Quality Control and Dataset Integration

Since interacting pairs of proteins tend to be expressed at broadly similar abundance ranges, global protein–protein interaction information can be used to derive an estimate of data quality for each dataset (8). For version 5.0 of PaxDb, all interacting protein pairs are retrieved from STRING v11.5 (38). For each dataset to be imported into PaxDb, the absolute log abundance ratios of interacting protein pairs are computed, and the median is taken. Then, the same operation is executed 500 times for the same dataset but with shuffled protein labels. The z-score of the observed median against the distribution of medians after label shuffling is termed “interaction z-score,” with a larger value indicative of better overall data quality.

In case more than one independent dataset is available for a given organism or tissue group, an “integrated” dataset is generated by weighted averaging. The estimation of the weights is iterative: the datasets are sorted by their interaction z-scores; the highest-scoring dataset receives a weight of 100%. Next, starting from the second-best to the lowest-scoring dataset, each new dataset is integrated with the previously merged datasets using ten equally spaced weights from 0 to 100%. The integration attempt with the highest score is selected.

#### Metadata Standards

Each dataset’s metadata, such as the organism name, taxonomy identifier (NCBI taxonomy (43)), tissue, tissue ontology ID, publication/source, PubMed ID, and quantification method (free text) are collected and made available *via* the website as well as the download files. Wherever possible, tissues are encoded with one of the following ontology systems: Uber-anatomy ontology (44), Plant Ontology (45), Cell Line Ontology (46), Cell Ontology (47), and The BRENDA Tissue Ontology (48).

### Human Proteome Comparisons

#### Data Collection

The Human Protein Atlas (HPA) normal tissue data based on version 21.1 and Ensembl version 103.38 was downloaded. Twenty-three tissues were subsequently mapped to their corresponding PaxDb tissue categories with identical labels for all but two tissues (“heart muscle,” “saliva gland” in HPA and HEART, SALIVA SECRETING GLAND in PaxDb, respectively).

Genotype-Tissue Expression (GTEx) proteome data were collected from Supplemental Table S2 (protein level) from (49). For each organ group, the GTEx consortium had quantified between 2 and 11 selected proteome samples per organ by mass spectrometry. The GTEx organ information of these samples was extracted and mapped, resulting in 13 PaxDb-matched organ groups. For the transcript expression data, gene-level transcripts per million values were downloaded from the GTEx portal from Analysis V8 (dbGaP Accession phs000424.v8.p2), involving 17,384 samples per organ. Fifteen organ groups were matched to PaxDb. For each organ group, the global means per gene across all samples was used.

#### Data Analysis

The HPA normal-tissue protein data were first filtered by the “Reliability” parameter: proteins in the “Uncertain” category were removed. The four protein levels: “Not detected,” “Low,” “Medium,” and “High” were then used for the comparison. A one-way ANOVA was performed for each tissue with PaxDb abundance ppm values, using HPA protein level as groups.

The PaxDb human proteome data (excluding GTEx experiments) were compared against GTEx RNA and protein datasets. A tissue-specific z-score was calculated for each gene and each tissue in PaxDb integrated data and GTEx data to represent relative protein expression (49). The Pearson correlation was calculated for z-scores between the tissue datasets. The Pearson’s correlation coefficients of organ groups from both PaxDb and GTEx were hierarchically clustered using average linkage to generate a heatmap.

### Proteome Evolution in Fungi

#### Orthology and Protein Domain Matching

One hundred seventy-nine *Fungi* species as well as five reference species from other Eukaryotic clades (*Homo sapiens*, *Drosophila melanogaster*, *Caenorhabditis elegans*, *Plasmodium falciparum*, and *Dictyostelium discoideum*) were included in the study. The “Simple Modular Architecture Research Tool” (50) was used to assign annotated domains to all encoded proteins in their genomes. Orthology relations between the genes in these species were retrieved from EggNOG version 5.0 (51). For all matched domain pairs between a reference species and a given Fungi species of interest, pairwise global sequence alignments were performed with EMBOSS-needleall (52) using the Needleman–Wunsch alignment algorithm (53). The domain pairs were filtered for at least 40% sequence identity, and only the highest-scoring alignment pair was considered in case multiple domains of the same type were annotated for any of the two orthologous sequences.

#### Amino Acid Usage Ratios Between Fungi and Reference Proteomes

For each orthologous protein pair, the amino acid usage was assessed within the aligned domain sections, and a ratio was computed. Only those orthologous pairs in which both proteins registered at least one amino acid of interest were considered. In cases where the orthology relation was complex (*i.e.*, where paralogous proteins are annotated in one or both of the organisms), the orthologous group was also not considered. For each species pair (Fungi species *versus* reference species), the protein abundance values were taken from the orthologous protein in *S.cerevisiae*, using its WHOLE ORGANISM integrated data. A Spearman’s rank correlation was then calculated between the amino acid usage ratios and protein abundances. For the visualizations in Evolution of Sulfur-Containing Amino Acids section, the data points were binned in six equally sized groups for the violin plots, and a linear regression was fit over all data points.

A taxonomy tree visualization was created for the 179 Fungi species according to the NCBI taxonomy database (43), using the interactive Tree Of Life (54) online tool. A heatmap was then added to the tree, visualizing the Spearman’s *ρ* values for each comparison between a Fungi species and a reference species.

## Results

### Data Update

**Fig. 1:**
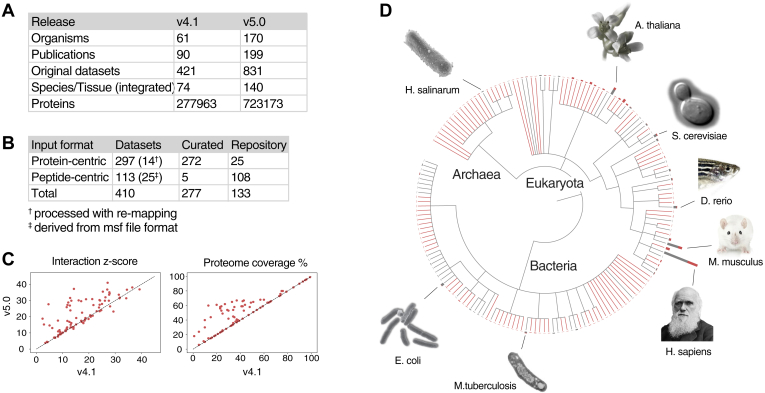
**PaxDb v5.0 data overview.***A*, comparison to the previous version (v4.1), in terms of number of organisms, publications, original and integrated datasets, peptide spectrum matches (PSMs), and proteins covered; *B*, origins and input formats of newly acquired datasets in v5.0; *C*, interaction consistency scores and proteome coverage for newly added as well as existing datasets. *D*, PaxDb 5.0 contains 170 species spanning three domains of life. *Gray lines* represent 61 species already existing in v4.1, and *red lines* represent 109 newly added species in v5.0. The associated bar plots indicate the number of datasets per species (*Gray*: existing; *Red*: new datasets). PaxDb, “Protein Abundances Across Organisms” database.

The current PaxDb version 5.0 has nearly doubled its data content with respect to the number of datasets, as well as the number of organisms covered (Fig. 1*A*). The PaxDb data integration pipeline involves a keyword-based discovery search for suitable projects and/or publications and automatic data processing for multiple input formats downloaded from repositories. Two hundred seventy-seven of the 410 newly imported datasets were derived from open-access publications’ supplementary files (“Curated”), and 133 were from data repositories (“Repository”). From a data processing point of view, 297 of the datasets were passed through our protein-centric import pipeline, which involves protein name mapping and where necessary also mapping *via* protein sequence comparison. In addition, 113 datasets were passed through the peptide-based import pipeline. Twenty-five of the latter were processed from msf files, and the rest from plain text inputs (Fig. 1*B*). Supplemental Figure S1 shows the dataset composition on three aspects using metadata from ProteomeXchange (for publication date and MS instruments) as well as processing software (from dataset-associated publication). The newly included original datasets were published between 2013 and 2021. The MS instrumentation was dominated by Q Exactive, followed by Orbitrap Fusion Lumos, LTQ Orbitrap Elite, LTQ Orbitrap, Orbitrap Fusion, and LTQ. Of the datasets for which published methods were available, the majority were processed using MQ or Proteome Discoverer.

Based on the protein interaction information derived from STRING v11.5 (38), we computed a quality score for each dataset (see Data Quality Control and Dataset Integration above). This “interaction consistency” score serves as a quality evaluation metric as benchmarked in (8). The integration pipeline relies on this score to weigh each dataset. Out of various weighting ratios, the dataset with the highest score is selected as the integrated dataset (*i.e.*, the best-estimate weighted combinations per species or tissue group). For example, the quality score and proteome coverage of 10 original human liver datasets range from 2 to 29.7 and from 12% to 52% respectively, while the human liver (integrated) dataset scores 30 with 80% proteome coverage.

Since genome annotations and name-spaces continually evolve, the datasets from version 4 of PaxDb also needed to be remapped and/or requantified. The scores were found to be mostly improved after the version update, when using the same STRING protein network as reference. For the integrated datasets, the interaction z-scores and proteome coverage nearly always increased when new datasets were added (see Fig. 1*C* for a comparison of for 93 integrated datasets with their counterparts in version 4). For existing integrations where no new datasets were added, the scores remained largely unchanged. However, with the inclusion of new datasets, the scores generally improved.

The 170 species in version 5.0 of PaxDb span the Archaea, Eukaryota, and Bacteria domains (Fig. 1*D*). The number of Archaea species increased from 1 to 22. While more species are included in Bacteria, Eukaryota encompasses a larger number of datasets owing to the diverse tissue-differentiated measurements. The top five species in terms of the number of datasets are *H. sapiens* (249), *A. thaliana* (59), *M. musculus* (106), and *D. rerio* (27). While ample datasets exist for these species and a few other model organisms, 104 species are represented by one dataset only (Supplemental Fig. S2*A*). On the species-tissue level of complex organisms, the most tissue groups exist in *H. sapiens* (64), followed by *M. musculus* (47), *A. thaliana* (15), and *D. rerio* (13). Also, 143 out of 235 species-tissue groups consist of only one dataset, while datasets accumulate in frequently studied tissues and species, for example human cell lines (61), human liver (11), and mouse liver (8) (Supplemental Fig. S2*B*).

*E. coli*, *H. sapiens*, and *B. subtilis* rank highest in proteome coverage, with *S. cerevisiae*, *P. falciparum*, *D. melanogaster*, *B. burgdorferi*, *H. pylori* 26695, *S. pombe*, and *M. musculus* trailing closely, each covering over 90% of their proteome (Supplemental Fig. S2*C*). In 63 out of 170 species, at least one dataset covers 50% proteome. On the species-tissue level of complex organisms, in 35 species-tissue groups, the proteome coverage exceeds 50%, most of them from *H. sapiens*, followed by *T. aestivum* root (77%), *A. thaliana* root (53%), and mouse cerebellum (51%) (Supplemental Fig. S2*D*).

### Comparison to Other Human Proteome Resources

To independently assess the validity of PaxDb data, it was compared to two comprehensive gene expression resources focusing on *H. sapiens*: the GTEx project as well as the HPA, both of which contain tissue-specific protein expression data.

#### Genotype-Tissue Expression

**Fig. 2:**
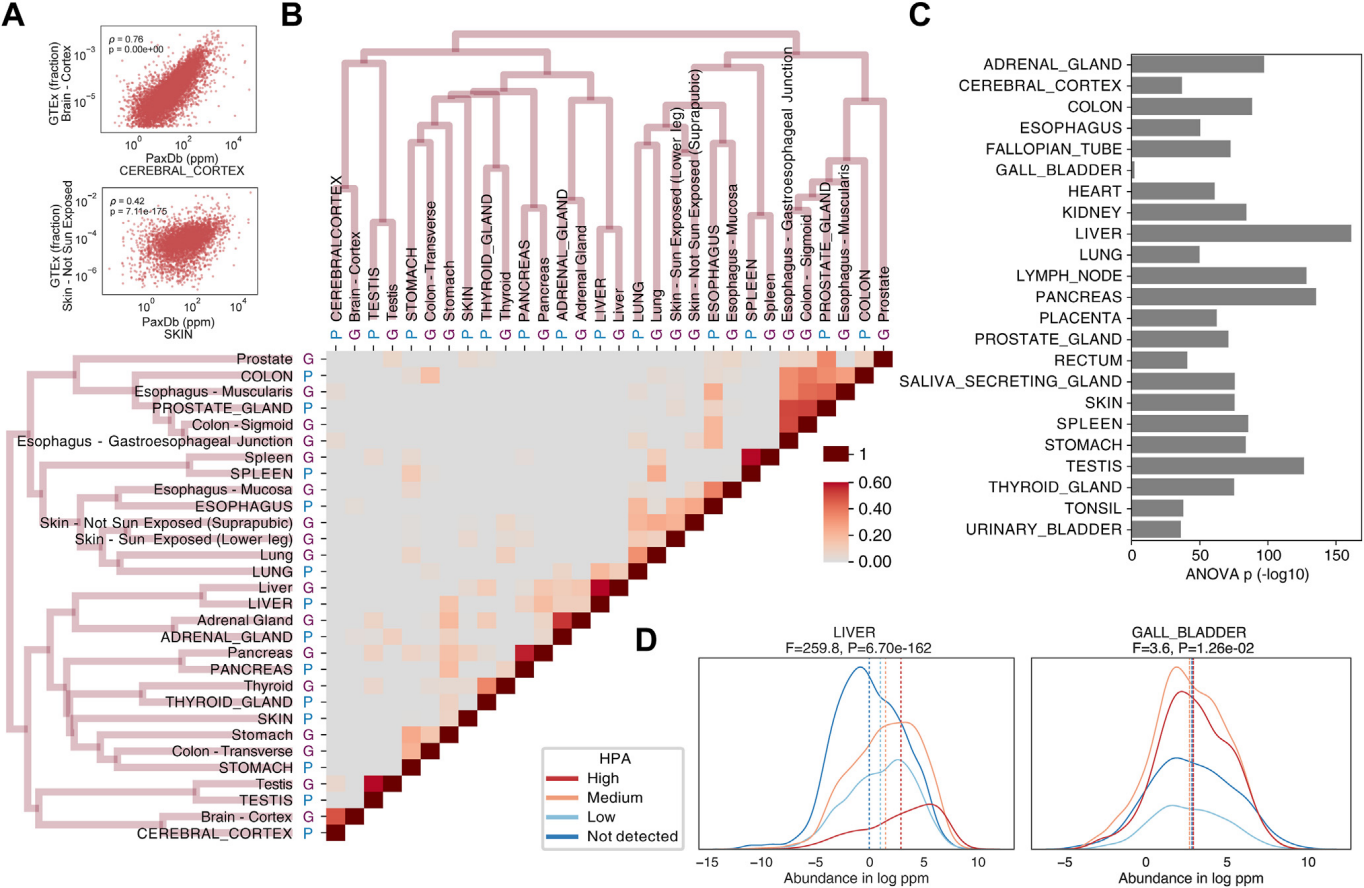
**Human proteome quantifications across distinct database resources.** PaxDb quantifications are compared to Human Protein Atlas and GTEx data. *A*, Spearman’s correlation between PaxDb abundances and GTEx protein abundances, shown for two example tissues: cerebral cortex and skin. *B*, Pearson’s correlation for tissue specific abundances in PaxDb against GTEx protein with clustering dendrogram, with PaxDb tissues marked with “P” and GTEx tissues with “G.” *C*, significance of abundance separation by HPA group label, per human tissue type. *D*, two examples of protein abundance distributions stratified by HPA categories (liver and gallbladder), showcasing the highest and lowest ANOVA *p*-values as depicted in *C*. GTEx, Genotype-Tissue Expression; HPA, Human Protein Atlas; PaxDb, “Protein Abundances Across Organisms” database.

Both RNA-level and protein-level data from GTEx samples were used. The PaxDb abundances and GTEx protein-level data were directly compared. For the matched labels, the Spearman’s correlation ρ ranged from 0.76 for cerebral cortex to 0.42 for skin (Fig. 2*A*). Due to the ubiquitous core biological processes, proteome-wide abundance has been observed to highly correlate across different tissue origins (55). Nevertheless, when comparing PaxDb and GTEx protein expression, the correlation coefficient ρ was higher for matching tissue pairs than nonmatching ones (Supplemental Fig. S3*B*). From PaxDb version 4 to 5, correlation increased regardless of whether the pairs were matching, due to the increased dataset score and proteome coverage (Supplemental Fig. S3, *B* and *C*). As nonmatching tissue pairs could still show correlation, particularly in cases where the tissues do not exhibit tissue-specific patterns, we use the tissue specificity z-score as described in (49) for the all-against-all comparison between PaxDb and GTEx in Figure 2*B*. The tissue specificity z-score was calculated per gene to represent a gene-level signature per organ group for both GTEx and PaxDb. The “signatures” from both resources were clustered by their pairwise Euclidean distance, and a heat map was colored by the Spearman correlation coefficients. At the protein level, 8 out of 13 matched organ group pairs between PaxDb and GTEx clustered together, including adrenal gland, brain cerebral cortex, lung, liver, pancreas, spleen, thyroid gland, and testis (Fig. 2*B*). At the RNA level, 9 out of 15 pairs clustered together, including adrenal gland, brain cerebral cortex, colon, esophagus (partially), fallopian tube, kidney, testis, stomach, and spleen (Supplemental Fig. S3*A*). The agreement between GTEx and PaxDb was stronger for protein data compared to RNA, despite the markedly smaller sample size in protein data. T tests for in-group and out-group Euclidean distances showed significance for both RNA (*p*-value 2.28 × 10^−2^) and protein (*p*-value 3.64 × 10^−5^). While over half of the organ pairs demonstrated similarity both at the protein and at the RNA levels, others did not. The discrepancy could be due to multiple tissue lineages within certain organs, such as the stomach, esophagus, and colon, which encompass epithelial and muscle layers. The proportions of these sampled tissues could influence the expression landscape. Certain organs, like the prostate, have previously been reported to be relatively indiscriminate (49). Furthermore, differences might also result from lower proteome coverage. For example, the skin dataset from PaxDb (excluding GTEx) only covered 23% of the proteome, which likely diminished its potential to distinguish tissue types.

#### Human Protein Atlas

While PaxDb data are almost entirely derived from MS data, the HPA normal tissue datasets approximate the protein expression profiles with antibody-based tissue microarrays. PaxDb computes protein abundance in continuous ppm values, while HPA reports protein expression in four levels, from “high,” through “medium” and “low”, to “undetected”. The different characteristics of either technology may result in systematic biases in the results, but a global abundance trend is expected to be observable in both. The overlapping proteins were grouped using HPA abundance labels, and the distribution of protein abundance based on PaxDb data was visualized using a kernel density plot, with a vertical line representing the mean of each group. Two examples of such plots are shown in Figure 2*D*. The level of correspondence between PaxDb protein abundances and the HPA abundance labels is reflected in the label groups’ separation as well as in their expected relative ordering from low to high. We analyzed the group differences with one-way ANOVA. Across the 23 tissues, the ANOVA *p*-values varied between 1.26 × 10^−2^ in GALLBLADDER and 6.7 × 10^−162^ in liver (Fig. 2, *C* and *D*). The order of protein groups ranging from “high” to “undetected” concurred with the PaxDb abundance group averages for all tissues, except for the GALLBLADDER and HEART.

### Evolution of Sulfur-Containing Amino Acids in Fungi

**Fig. 3:**
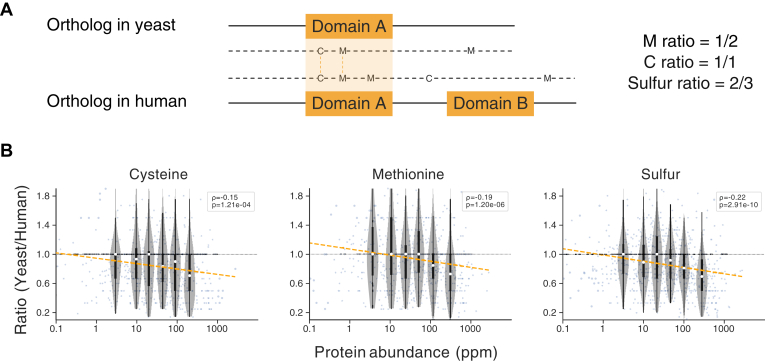
**Comparing sulfur usage in orthologous protein pairs.***A*, to avoid potentially confounding effects of evolutionary changes in protein function, only equivalent and alignable protein domains within strict 1-to-1 orthologs are considered. *B*, relations between protein abundance and the sulfur-usage ratio yeast/human. Each data point corresponds to one orthologous pair of proteins; the abundance values on the x-axis are taken from the yeast protein. Violin plots indicate median and percentiles 25 and 75, for six equally sized bins. A linear regression was fit for the regression line. Spearman’s *ρ* and its *p*-value were separately derived.

The relative frequencies of amino acids in the overall proteome can change at evolutionary timescales (56), and they are known to differ across organisms in response to mutational and environmental processes (57, 58, 59). While inspecting datasets in PaxDb, we noticed that S-containing amino acids (cysteine and methionine) seemed to be markedly underrepresented in the proteomes of certain Fungi. On its own, this observation is difficult to interpret: it could be the consequence of distinct functional compositions of these proteomes (*e.g.*, unusual fractions of secreted proteins) or the result of genome-wide mutational biases, or it could suggest an adaptive response. To narrow down potential causes, we compared Fungi proteomes to a varied set of other eukaryotic proteomes and restricted the comparisons to strictly one-to-one orthologous gene pairs (Fig. 3). In addition, we further restricted the analysis to functionally and structurally equivalent parts within these orthologs (*i.e.*, aligned protein domains); doing so should largely cancel out effects caused by differences in overall proteome functions. Then, to distinguish genome-wide mutational effects (*e.g.*, G/C content differences) from potentially adaptive effects, we further stratified proteins by their absolute abundance levels—adaptive changes in response to sulfur limitations should be visible particularly in highly expressed proteins.

We systematically conducted this analysis on the proteomes of 179 Fungi species, for which the encoded proteomes, protein domain compositions and orthology relations have been established (38, 50, 51). We compared their sulfur usage against five representative eukaryotes from other clades, selected for high proteome coverage in PaxDb. The latter included the human, two animal model organisms (*C. elegans* and *D. melanogaster*) and two unicellular eukaryotes (*P. falciparum* and *D. discoideum*).

When comparing the ratios of S-containing amino acids across orthologous gene pairs, we observed for the majority of proteins expressed at low to medium levels, that the overall usage of sulfur was roughly similar (*i.e.*, the median ratio centered on 1.0, see Fig. 3). However, remarkably, this ratio dropped below 1.0 for more strongly expressed, abundant proteins. This trend is highly significant and is observed independently both for cysteine as well as for methionine. It also made no difference whether protein abundance levels were taken from one or the other of the two organisms (not shown); the yeast (*S. cerevisiae*) was taken as a reference for protein abundances for all subsequent analysis because of its highest quality and coverage in PaxDb, within the Fungi clade.

**Fig. 4:**
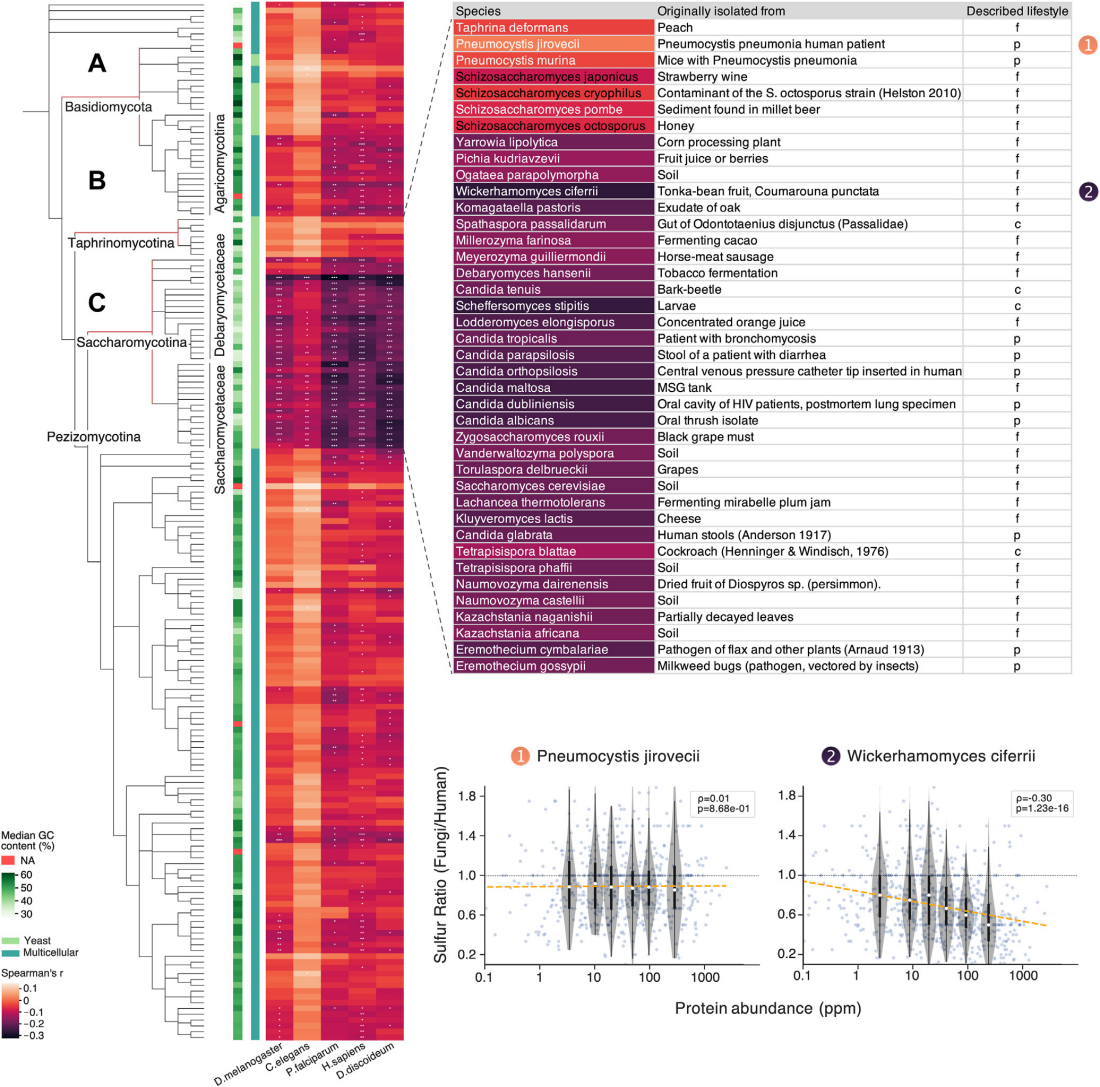
**Patterns of reduced sulfur content in Fungi proteomes.** The proteomes of 179 Fungi (*rows*) are compared to the proteomes of five reference organisms from other Eukaryotic clades (*columns*). Each tile in the heatmap indicates the strength of the negative correlation (Spearman’s *ρ*) between protein abundance and the sulfur-usage ratio Fungi/Reference. *Asterisks* indicate the significance (*p*-value) of the correlations: ∗: <0.01, ∗∗: <0.001, ∗∗∗: <0.00001. Each Fungi’s genomic GC-content and multicellularity status are color-labeled on the left of the heatmap. The Fungi are arranged according to their taxonomic annotations at NCBI (43). The Taphrinomycotina (*B*) and Saccharomycotina (*C*) clades are shown expanded to the right, together with the heatmap colors of the Fungi/human comparisons; the inset includes information on the environment from which the original type strains were collected. Two Fungi species from the table (*P. jirovecii* and *W. ciferri*) are marked; their detailed correlation data are shown in the inset below, similar to Figure 3.

Assessing the strength of this effect across all 179 Fungi, a differential sulfur depletion pattern was observed (Fig. 4). Separate heatmaps for cysteine and methionine shows coordinated regulation patterns (Pearson’s r ranges between 0.52 and 0.74 against five reference species; Supplemental Fig. S4 and Supplemental Table S1). While the majority of Fungi species showed at least some degree of sulfur avoidance against the five reference species, the reduction was the strongest in the *Saccharomycotina* order (C in Fig. 4), containing baker’s yeast as well as most other unicellular Fungi species (*i.e.*, yeasts). Assuming that the observed sulfur avoidance in the *Saccharomycotina* might be adaptive, *i.e.*, perhaps related to recurring episodes of sulfur limitation, a multicellular lifestyle would have been advantageous as it could provide mobility to escape the limiting environments. In the Fungi kingdom, the marker for multicellularity is the development of hyphae, long tubular substrate-seeking extensions, which allow the organisms to survive and migrate away from nutrient-poor areas (60). We asked whether other unicellular yeasts besides *Saccharomycotina* showed elevated sulfur avoidance. As the unicellularity in Fungi is not monophyletic, we marked the unicellularity/multicellularity of the species in Figure 4 according to MycoBank (61). Although organisms in the *Taphrinomycotina* subdivision (B in Fig. 4) and a few species in the *Basidiomycota* division (A in in Fig. 4) are also unicellular, their proteomes did not exhibit similar levels of sulfur reduction as the *Saccharomycotina*. Subsequently, we explored whether the observed differences could be accounted for by the GC content. We obtained the median genome-wide GC% for each fungal species from NCBI Genome. When considering all species, there appeared to be a positive correlation between GC% and the sulfur ratio (Pearson’s r 0.38, *p*-val 2.3 × 10^−7^). However, upon excluding the *Saccharomycotina* clade, the correlation disappeared entirely (Pearson’ r 0.01, *p*-val 0.9). This indicates an associated effect of the lower GC content of *Saccharomycotina* clade. We further investigated the environment/host associations of the *Saccharomycotina* and the closely related *Taphrinomycotina* species. Habitat and/or lifestyle information for Fungi is not systematically available; we approximated it by the annotated sources of the first isolation of the type strains, as described in ATCC (https://www.atcc.org, accessed February 20, 2023); we also marked potential symbiotic lifestyles with “c”, parasites with “p”, and free-living lifestyles with “f”. For *Taphrinomycotina* (showing little sulfur avoidance), two out of six species were parasitic, while for *Saccharomycotina* (strong sulfur reduction), 11 out of 33 species were symbionts, six of which were parasitic. Overall, a clear association of sulfur avoidance with free-living or parasitic lifestyles was not observed. As the environments of the isolated type strain cannot fully represent the major habitat of the species, the underlying cause of the clade-wide sulfur avoidance was not established.

## Discussion

This update of PaxDb v5.0 reports a nearly three-fold increase in the number of species covered, and a two-fold increase in the number of original datasets and publications. Decreased evolutionary distances between the species will enable higher-resolution cross-species comparisons. Likely due to general improvements of the genome/proteome reference annotations, the re-mapping of older datasets from the previous version of PaxDb mostly resulted in dataset quality improvements.

Using two independent human-centered data collections—HPA and GTEx, we verified the overall data quality of PaxDb, in terms of quantitative agreement and dataset tissue matching. We compared the PaxDb integrated tissue-level protein abundances with matched GTEx RNA and protein abundances. While both showed strong correlation between the matched labels, the protein-level comparison contained fewer indiscriminate correlations and more in-group correlations than the RNA-level. Comparisons with antibody-based protein abundance estimates from HPA reached qualitatively similar conclusions. Using protein–protein interaction data as another, independent arbiter of data quality, we show that the PaxDb integration of multiple lower-coverage, lower-quality datasets enhances the data quality and provides a boost to overall proteome coverage.

By integrating PaxDb data with sequence analysis of orthologous protein pairs, we discovered an apparent, strong selection pressure to reduce sulfur usage in abundantly expressed proteins, in a particular clade of single-celled Fungi. One of the conceivable selection pressures causing this effect would be a recurring sulfur limitation in the environment. Experimentally induced sulfur depletion was shown to trigger an alternative proteome state, resulting in 30% reduction in sulfur usage in Fungi (62) and 45% reduction in a green alga, *Chlamydomonas reinhardtii* (63). Besides such transient responses to acute sulfur limitations in the environment, more prolonged sulfur limitation may also have resulted in adaptive changes in the genome. Baudouin-Cornu *et al.* (64) showed, for example, that sulfur assimilatory enzymes in yeast and *E. coli* are themselves encoded using remarkably little cysteine and methionine. Comparison of Cyanobacteria strains isolated from sulfur-rich and sulfur-poor environment showed adaptive eradication of cysteine and methionine in phycobilisome, the light-harvesting proteins and the major cellular component, in response to sulfur depletion (65). Another possible selective pressure against sulfur usage relates to oxidative toxicity. Unwanted disulfide bonds may be formed under oxidative stress, impacting protein folding and activity (66). If the organisms were exposed to oxidative stresses through their evolution, it could explain the reduction of cysteine (but not methionine) in their protein sequences.

However, why the *Saccharomycotina* in particular would show a reduced use of sulfur in their proteome remains unclear. The habitat ranges and ecological strategies of many Fungi are described only anecdotally, and even less is known about any present or past episodes of sulfur limitations. Nevertheless, Fungi are known to be able to assimilate sulfur from a number of sources, both of biotic and abiotic origin (67). Perhaps this diversity of assimilatory toolkits is a sign for past episodes of sulfur limitation. Future growth in the availability of genome sequences will allow this phenotype to be mapped with ever increasing resolution.

## Data Availability

The PaxDb database is freely accessible at https://pax-db.org. Database records from the previous versions are available at https://pax-db.org/downloads. Creative Commons Attribution 4.0 International (CC BY 4.0) applies to all content of PaxDb resource.

## Supplemental data

This article contains supplemental data.

## Conflict of interest

The authors declare no competing interests.
