# Supplementary material for: PaxDb 5.0: Curated Protein Quantification Data Suggests Adaptive Proteome Changes in Yeasts
Source: Mol Cell Proteomics. 2023 Aug 31;22(10):100640. doi: 10.1016/j.mcpro.2023.100640 (PMC10551891; doi:10.1016/j.mcpro.2023.100640)

Supplementary Material

Table of Contents

[Supplementary Figure 1: Descriptive statistics on the composition of newly included datasets in PaxDb v5.0 in terms of year of publication, processing software and instrument used for the measurements. 2](#_Toc143175013)

[Supplementary Figure 2: PaxDb datasets statistics. A: Number of datasets on species level. B: Number of datasets on species-tissue level. The top right of A and B panels show cumulative number of datasets per groups (species for A, species-tissue group for B). The dashed line indicates the slope in case of one dataset per group. C: Proteome coverage on species level. D: Proteome coverage on species-tissue level. 3](#_Toc143175014)

[Supplementary Figure 3: A: Pearson's correlation of tissue specificity z-scores in PaxDB against GTEx RNA expression data with clustering dendrogram, with PaxDB tissues marked with “P” and GTEx tissues with “G”. B: Spearman correlation of PaxDb and GTEx protein expression for PaxDb version 4 and 5 for the matched and not matched tissue pairs. C: For the matched tissue pairs, the scatter plot of the change in Spearman’s ρ against the change in dataset score and against the change in proteome coverage. 4](#_Toc143175015)

[Supplementary Figure 4: The proteomes of 179 Fungi (rows) are compared to the proteomes of five reference organisms from other Eukaryotic clades (columns) with respect to cysteine (left), methionine (middle) and sulfur. 5](#_Toc143175016)

[Supplementary Table 1: Sulfur avoidances across all fungi, comparing the effects between cysteine and methionine. Pearson’s correlation of correlations (Spearman’s ρ shown in Fig. S4: usage ratios vs. abundance) of all fungi, separately for five reference species. 6](#_Toc143175017)


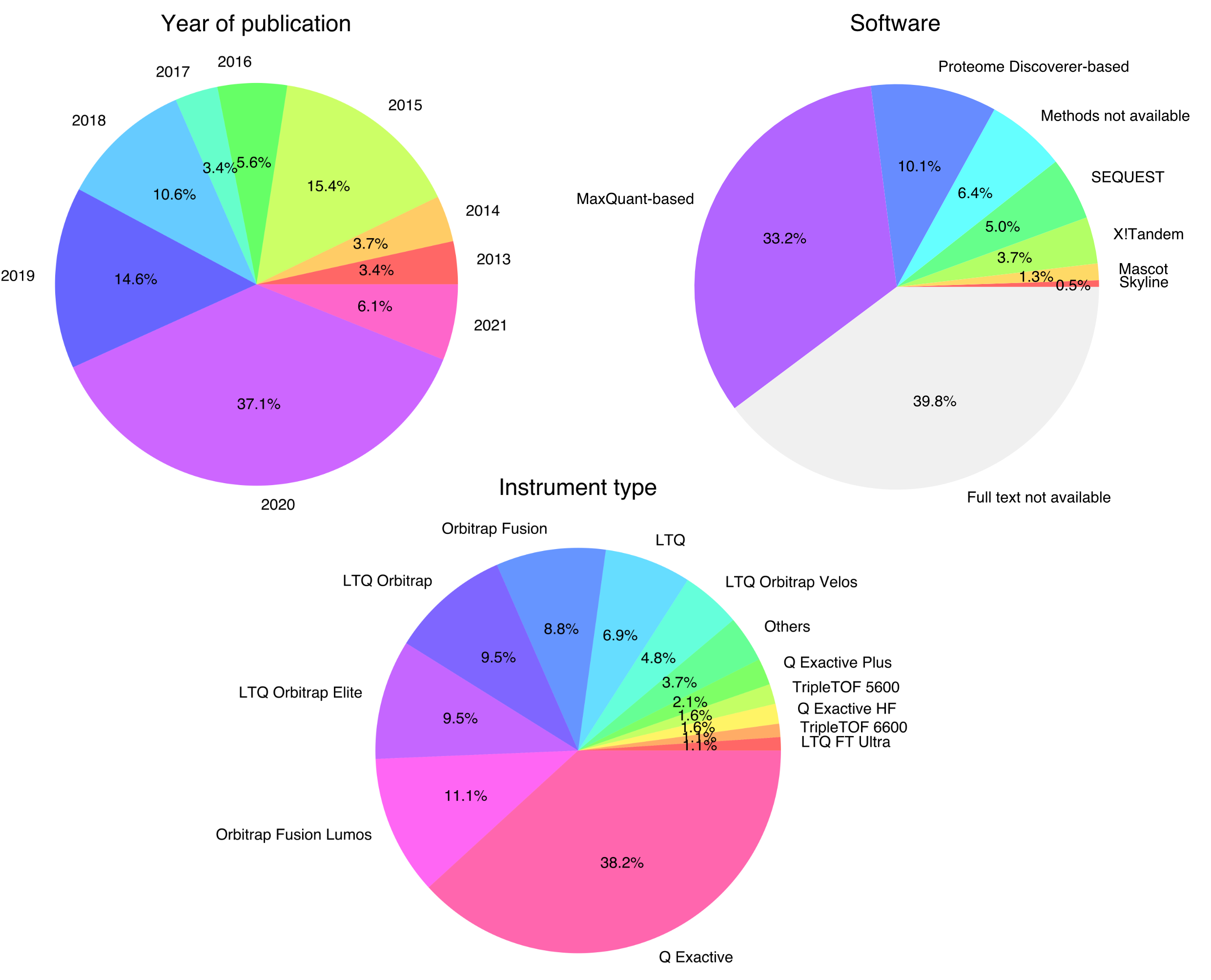


# Supplementary Figure 1: Descriptive statistics on the composition of newly included datasets in PaxDb v5.0 in terms of year of publication, processing software and instrument used for the measurements.

­­­­
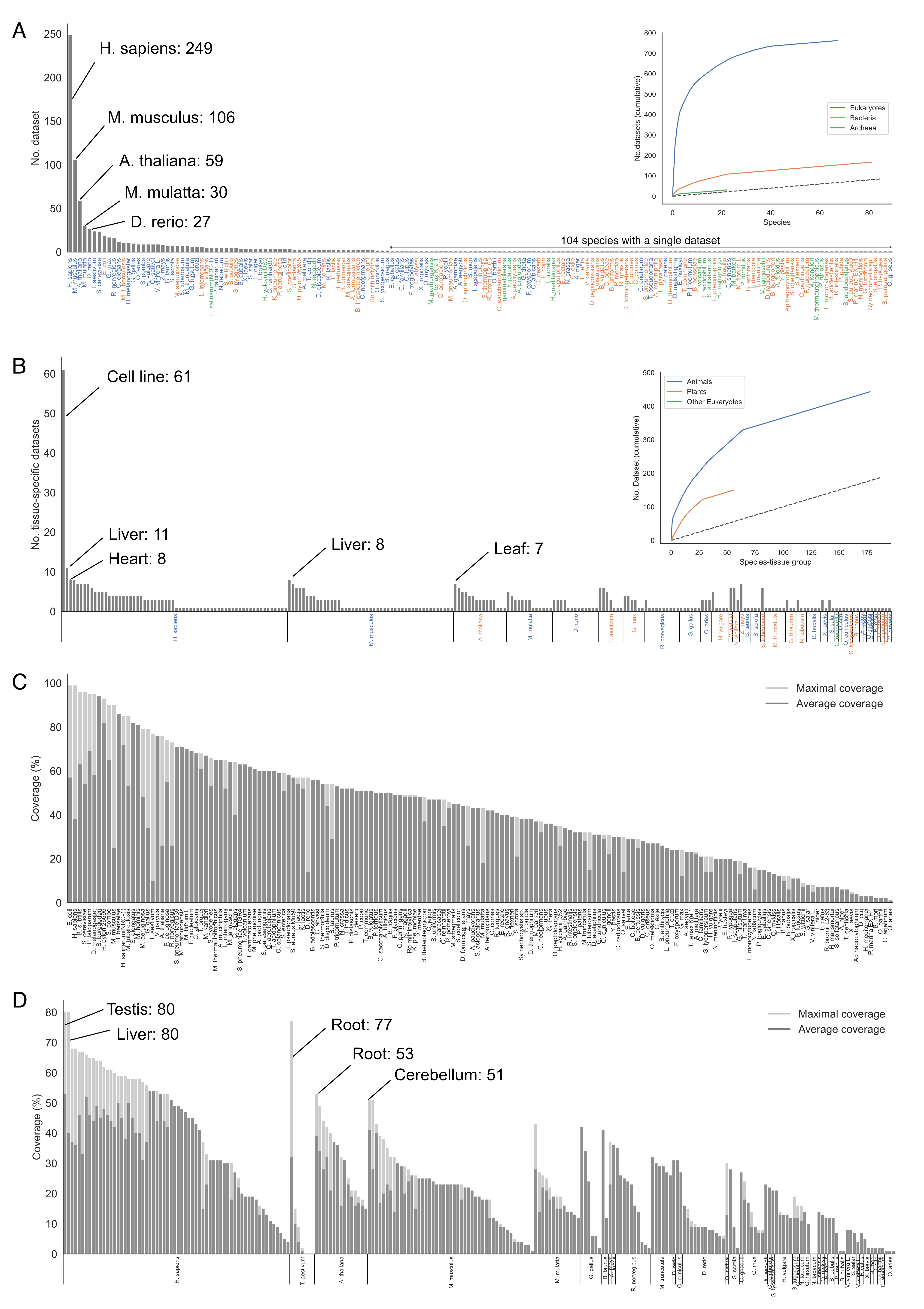


# ­­­­­Supplementary Figure 2: PaxDb datasets statistics. A: Number of datasets on species level. B: Number of datasets on species-tissue level. The top right of A and B panels show cumulative number of datasets per groups (species for A, species-tissue group for B). The dashed line indicates the slope in case of one dataset per group. C: Proteome coverage on species level. D: Proteome coverage on species-tissue level.


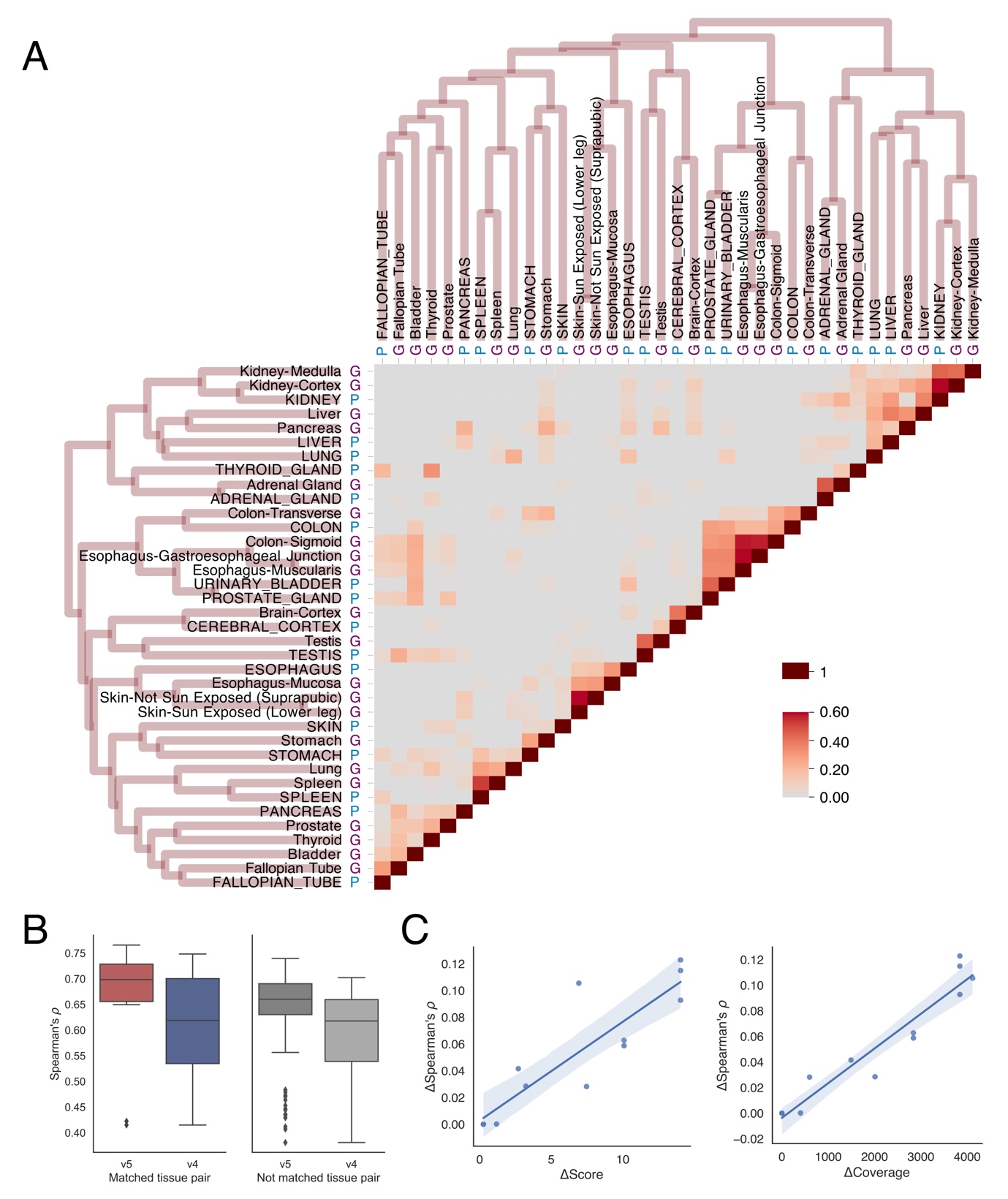


# Supplementary Figure 3: A: Pearson's correlation of tissue specificity z-scores in PaxDB against GTEx RNA expression data with clustering dendrogram, with PaxDB tissues marked with “P” and GTEx tissues with “G”. B: Spearman correlation of PaxDb and GTEx protein expression for PaxDb version 4 and 5 for the matched and not matched tissue pairs. C: For the matched tissue pairs, the scatter plot of the change in Spearman’s ρ against the change in dataset score and against the change in proteome coverage.

# Supplementary Figure 4: The proteomes of 179 Fungi (rows) are compared to the proteomes of five reference organisms from other Eukaryotic clades (columns) with respect to cysteine (left), methionine (middle) and sulfur.

Each tile in the heatmap indicates the strength of the negative correlation (Spearman's ρ) between protein abundance and the sulfur-usage ratio Fungi/Reference. Asterisks indicate the significance (p-value) of the correlations: *: < 0.01, **: < 0.001, ***: < 0.00001.

# Supplementary Table 1: Sulfur avoidances across all fungi, comparing the effects between cysteine and methionine. Pearson’s correlation of correlations (Spearman’s ρ shown in Fig. S4: usage ratios vs. abundance) of all fungi, separately for five reference species.


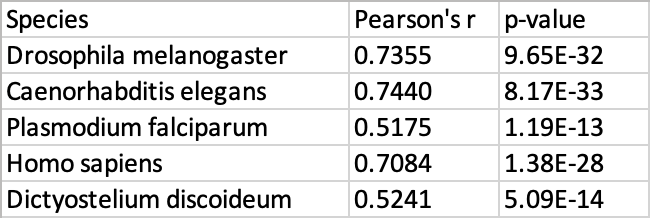

Supplement: Supplemental data [file mmc1.docx]
